# “It is always better for a man to know his HIV status” – A qualitative study exploring the context, barriers and facilitators of HIV testing among men in Nairobi, Kenya

**DOI:** 10.1371/journal.pone.0231645

**Published:** 2020-04-15

**Authors:** Jerry Okal, Daniel Lango, James Matheka, Francis Obare, Carol Ngunu-Gituathi, Mary Mugambi, Avina Sarna

**Affiliations:** 1 Population Council, Nairobi, Kenya; 2 Nairobi City County, County Health Services, Nairobi, Kenya; 3 National HIV and STI Control Programme (NASCOP), Nairobi, Kenya; 4 Population Council, New Delhi, India; Johns Hopkins Bloomberg School of Public Health, UNITED STATES

## Abstract

HIV testing services are an important component of HIV program and provide an entry point for clinical care for persons newly diagnosed with HIV. Although uptake of HIV testing has increased in Kenya, men are still less likely than women to get tested and access services. There is, however, limited understanding of the context, barriers and facilitators of HIV testing among men in the country. Data are from in-depth interviews with 30 men living with HIV and 8 HIV testing counsellors that were conducted to gain insights on motivations and drivers for HIV testing among men in the city of Nairobi. Men were identified retroactively by examining clinical CD4 registers on early and late diagnosis (e.g. CD4 of ≥500 cells/mm, early diagnosis and <500 cells/mm, late diagnosis). Analysis involved identifying broad themes and generating descriptive codes and categories. Timing for early testing is linked with strong social support systems and agency to test, while cost of testing, choice of facility to test and weak social support systems (especially poor inter-partner relations) resulted in late testing. Minimal discussions occurred prior to testing and whenever there was dialogue it happened with partners or other close relatives. Interrelated barriers at individual, health-care system, and interpersonal levels hindered access to testing services. Specifically, barriers to testing included perceived providers attitudes, facility location and set up, wait time/inconvenient clinic times, low perception of risk, limited HIV knowled ge, stigma, discrimination and fear of having a test. High risk perception, severe illness, awareness of partner’s status, confidentiality, quality of services and supplies, flexible/extended opening hours, and pre–and post–test counselling were facilitators. Experiences between early and late testers overlapped though there were minor differences. In order to achieve the desired impact nationally and to attain the 90-90-90 targets, multiple interventions addressing both barriers and facilitators to testing are needed to increase uptake of testing and to link the positive to care.

## Background

Kenya is among the countries with exceptionally high HIV epidemic in the world (alongside Mozambique and Uganda) with 1.6 million people living with HIV in 2016 and ranked fourth globally [1, 2]. Overall, the HIV epidemic in Kenya is driven by sexual transmission and is generalized among all sections of the population including children, young people, adults, women and men [3]. Whereas a disproportionate number of new infections occur among specific population groups such as female sex workers, people who inject drugs, men who have sex with men, men in the general population also carry a considerable burden of HIV infection with approximately 4 percent estimated to be living with HIV. HIV testing services (HTS) is a major feature of Kenya’s HIV response [4] as it offers timely linkage to care and treatment, prevention of onward transmission to sexual partners and reduction of risky behaviors [5–8]. However, major challenges in the HIV care cascade remain for men [9–18]. Recent population-based evidence from Kenya demonstrated that, 53% of women had tested for HIV in the past 12 months and received their results, compared to 45% of men [19]. Further, it is estimated that 35% of all people living with HIV in Kenya are men and that approximately 33% of new HIV infections occur among male population [20, 21]. Similar patterns have been observed in other regions of sub-Saharan Africa (SSA) where few men test and access HIV services late in the disease process [18, 22–25].

Men are less likely to access health services in a timely manner compared to women due to several factors which by extension can limit HIV testing coverage. Existing evidence suggests a variety of possible barriers to uptake of HIV testing among men in SSA including individual/personal factors such as gender norms [26], fear of results [16–18], stigma and discrimination [14, 19], low perception of risk [2, 15, 20], and fear of disclosure. Facility-related factors such as provider attitude and (lack of) confidentiality [27], inadequate supplies and equipment [28] have been documented to hinder access to testing services [29, 30]. Diverse structural factors such as sub-optimal targeting, inadequate public information have also been noted to obstruct access to HIV testing [28]. In addition to these barriers, HIV testing is a key defining event for most people because of stigma and fear and uncertainty associated with a positive result [31, 32]. Hence, in promoting HIV testing uptake, a vital question is how to position testing and the potential negative effect of learning one has HIV against the benefits of learning one’s status [31]. Part of the UNAIDS 90-90-90 strategy to counter some of the barriers to testing outlines the importance of reducing HIV-related stigma, addressing the fears of testing, promoting HIV testing, social norms, providing options for rapid and non-blood-based HIV tests, and ensuring non-judgmental and culturally competent HIV counselling and testing services [13, 33–36]. Yet, with these vibrant strategies, data suggests that neither testing nor initiation to treatment has reached optimal status in SSA [22, 23, 37, 38], and specifically among men in the region [14, 17, 39–41]. There is therefore a need for interventions to enhance HIV testing coverage for men to help with identification of new HIV cases and early initiation into care. Formulating such interventions requires an understanding of the context of testing as well as existing barriers and facilitators to HIV testing from the perspectives of both men and health providers.

While there is some regional evidence and information on the context, barriers and facilitators to HIV uptake among men, such evidence is severely limited or nonexistent in Kenya. The few studies on HIV testing status among men in Kenya largely focus on their role in the Prevention of Mother to Child Transmission (PMTCT) services or oral self-testing and less on men as HIV testing clients. The updated HIV testing and treatment guideline in Kenya recommend expansion of HIV testing through self-testing and partner notification, provider initiated testing (PITC) in addition to rapid initiation of ARVs [42]. Given the low uptake of HTS among men, there is a need for evidence on factors surrounding HIV testing to better understand the motivations and drivers of HIV testing among this segment of the population. This study was anchored a sociological concept “positive deviance” (PD), that aims to encourage desirable behaviors by learning from individuals who are deviant in a positive sense (Marsh et al., 2004). Thus, the selection of HIV positive men in this study was based on the need to understand their motivations to test (and challenges) and determine the unique attributes or strategies that enable them test than their peers who are untested or HIV negative, despite facing similar challenges. Using qualitative data, this paper presents an in-depth understanding of the context, perceived barriers and facilitators of HIV testing among men who tested early or late in an urban setting in Kenya. We also sought provider’s perspectives on men’s uptake of HTS to document providers views on health facility and structural factors and health seeking behaviors of men accessing services at the facilities. A highly nuanced understanding of men’s experiences with HIV testing can inform the design of targeted interventions aimed at improving the uptake of HIV services for specific population sub-groups.

## Methods

### Study design

This was a cross-sectional qualitative study that was conducted as part of USAID-funded Project SOAR portfolio entitled ‘Improving Uptake of HIV Testing Services and Linkage to Care after Diagnosis in Kenya’ conducted in 2018 to understand the motivations and drivers for getting tested. The study was implemented in two phases. In Phase 1 we implemented a mixed method, cross-sectional study that entailed (a) scoping of HTS sites; (b) surveying men seeking HIV testing at HTS centers; and (c) qualitative in-depth interviews (IDIs) with HIV-positive men and counsellors providing HTS services. The objective of the study was to identify site-specific characteristics that contribute to attracting male clients for HIV testing; to examine men’s knowledge, attitudes, and practices around risk behaviours and HIV testing; and to understand their motivations and drivers for getting tested. In Phase 2, we developed and evaluated a simple post-test assessment tool for HTS (SPAT) for counsellors to use to improve HTS services [43].

As part of Phase I activities IDIs were conducted with HIV testing counsellors (HTC) and men living with HIV (MLHIV) who were on care in eight health centers. The participating health facilities were selected based the number clients accessing HIV testing services. Selection of facilities based on the number of clients accessing HTS and geographical location was done to try and determine whether access to services by men is enhanced or impeded the facilities client load and location. Existing literature has documented stigma and discrimination, confidentiality and long wait time as barriers to HTS services [14, 19, 27]. We conducted a mapping and scoping of health facilities providing HTS services in Nairobi County. A total of 124 out of 381 facilities providing HTS in Nairobi representing the four tiers of health care service delivery in Kenya were purposively selected based on the following characteristics: client load (high versus low volume), total number of males versus female testers, catchment area and HTS type (i.e., sites where antiretroviral therapy is offered versus standalone HTS sites). The services statistics data was used to categorize centers with high- and low client load. We classified High volume HTS sites (HMV) as sites that had ≥45% of clients accessing HTS being male, while Low Male Volume (LMV) categorized as facilities with less than 45 percent of clients accessing HTS services being male. This cut-off was based on the evidence that more women (80%) than men (62%) were likely to be tested for HIV [44]. The health facilities serve patients, families, communities, and key populations and they cover emergency, preventative, hospital, diagnostic, and primary care. Since the promulgation of the new Kenyan constitution in 2010 management of these health facilities shifted from central government to the City County government of Nairobi. The IDIs focused on reasons for testing early or late, knowledge of HIV transmission, perceived risk, risk behaviors, stigmatizing attitudes towards people living with HIV, gender norms, time taken and process of arriving at a decision to test, persons/peers consulted, and expectations prior to testing and preparedness for the result.

The study protocol was reviewed and approved by institutional review boards of the Population Council and Kenyatta National Hospital/University of Nairobi ethics and research committee and National Commission for Science, Technology & Innovation (NACOSTI). Participants were given a modest reimbursement of Ksh 300 ($3) to cover their transport costs.

### Participant recruitment

#### Recruitment of providers

The HIV testing counsellors who were directly involved with testing services in eight study sites were purposively selected following consultation with facility in-charges. Eligibility criteria for participation in the in-depth interviews for the counsellors included: being aged 18 years or older, proficiency in Kiswahili or English, participation in the HIV testing program at least 4 months prior to the interview, and ability/willingness to provide written consent.

#### Recruitment of Men Living with HIV (MLHIV)

MLHIV who had received their positive diagnoses and accompanying CD4 counts six to 12 months prior to recruitment were identified retroactively by examining clinical records and identifying 15 men whose CD4 count was ≥500 cells/mm around the time of diagnosis (i.e., early diagnosis) and 15 men whose CD4 counts were <500 cells/mm at the time (i.e., late diagnosis). We recruited approximately 3–4 MLHIV from each of the eight facilities. Other eligibility requirements for the MLHIV included being aged 18 years or older, proficiency in Kiswahili or English, ability/willingness to provide written consent and residing in the study area (Nairobi City County) for at least 1 month prior to the interview. The counselors obtained client information, contacts, provided a brief explanation of the study and invited them to visit the facilities and participate in the interview. Overall, the refusal rates were below 5%.

### Data collection

In-depth interviews were conducted by study team members trained in qualitative data collection, with each facilitator recording the conversation and taking short notes when needed. All participants provided information on age, marital status, education, occupation, duration of knowledge of HIV status, and whether they were residents of Nairobi. Interviews lasted approximately 40–60 minutes and were conducted in Kiswahili, English or a mixture of both depending on participants’ preference. All discussions were digitally recorded, transcribed and translated into English.

Efforts were made to ensure confidentiality during the study. Participants’ names or any other identifying information were not recorded, and the interviews were conducted in private settings (e.g. designated safe spaced within the facility setting)–away from audio and visual disturbance.

### Data analysis

Qualitative interviewers wrote initial impressions of key findings, observations and issues for further exploration before transcriptions were done. Following transcriptions, two qualitative analysts (JO and DL) first read through the transcripts independently to identify broad themes and generate descriptive codes and categories. The codes were then compared, to resolve discrepancies through discussion and consensus and a final code list was developed. The analysis was conducted iteratively and focused on the context, barriers and facilitators to HIV-testing.

## Results

### Socio-demographic characteristics of study participants

We conducted in-depth interviews with 30 MLHIV who received a positive diagnosis within a period of 6–12 months, and eight HIV testing counselors directly involved with the provision of testing services at eight facilities in Nairobi.

#### MLHIV

The mean age of all MLHIV was 34.2 years (SD: 10.16). Most participants were married (67%), were residents of Nairobi city, had some primary (43%) or secondary education (33%) and were employed (70%)–most in the informal sector (Table 1).

**Table 1:** Socio-demographic characteristics–MLHIV.

Characteristics	N = 30 n (%)
**Current Residence**	
Urban	30 (100)
Age—Mean (range), SD	34.23 (20–54) SD 10.16
**Marital status**	
Married	20 (66.7)
Single	6 (20.0)
Widowed	3 (10.0)
Separated	1 (3.3)
**Education Status**	
Primary	13 (43.3)
Secondary	10 (33.3)
College–mid level	5 (16.7)
College–University	2 (6.7)
**Employment status**	
Formal	5 (16.7)
Informal	16 (53.3)
Unemployed	9 (30.0)

#### Counsellors

The mean age of all counsellors was 36 (SD 6.74) and the mean number of years in service was 6.1 with a standard deviation of 4.00. All participants had minimum “O” level secondary education as well as HIV counselling training. Most counsellors were females (6/8).

### The context of HIV testing

In this section we present data on the context leading to HIV testing among MLHIVs including timing of conducting the test, decision-making process including people consulted (e.g. support received from peers, friends and relatives) and the actual HIV testing experience as these can provide understanding of the decision-making processes which can inform male-targeted interventions. Overall, as previously noted we documented minor differences in the experiences of HIV testing among early and late testers.

#### Time taken to arrive at a decision to test

While some men (n = 7/30) took a short time (a week or less) to test after making up their mind to test, most (23/30) took one or more months to test. Participants who tested early had strong social support systems, a supportive partner, and agency to test.

“*So*, *I talked with my wife and we finished that issue (regarding testing) and then she encouraged me to go for testing*, *I then went to the facility and got tested the next day* (MLHIV, 27 years, Married)

Decision to test after one or more months was often linked to concerns around cost of services, stigma and poor partner relations. Among the specific reasons given for testing late were cost of conducting an HIV test and scouting for facilities which were far away from place of residence/work.

*The hospital is far from where I live and sometimes when people know you are infected*, *they see you as useless*. *And as I was explained even if we meet on the streets*, *they won’t expose me*, *we will just exchange greetings and life goes on*. *I was assured that I can come for medication or treatment any time*. *(MLHIV*, *27 years*, *married*)

Indecision to test and poor inter-partner dynamics prolonged the decision and timing to test (for example tension with partners arising from “poor communication and lack of transparency in the relationships”). Participants were reluctant to initiate discussions with their partners for fear of backlash. Therefore, some participants used a variety of strategies (both covert and overt) to initiate discussion to test.

*“It took me a long time*. *We had been talking with my wife and she always confidently told me she and the children are okay*. *It took me about two weeks*. *I first saw the card (*referring to the card normally used to access HIV-related services*) and ignored and since I didn’t want to make her angry*, *I waited for some time*. *When I eventually asked her*, *she responded that she is negative and her children*.” *(MLHIV*, *37 years*, *married*)

#### Consultations and actions taken prior to testing

Participants were asked whom they talked to or sought advice from before testing for HIV and overall (across both early and late testers), minimal consultations were made prior to testing although whenever discussions occurred, it was with partners or other close relatives who offered support to test.

*Yes*, *my brother*, *he works with (Facility name) Hospital*. *He accompanied me on his way to work*, *and encouraged me to go and get tested*, *and told me of case scenarios at his place of work*, *since he worked at a hospital*, *he saw several people who were living with HIV and went about their business normally*. *(MLHIV*, *40 years*, *married*)

In view of minimal communication prior to testing, nearly all participants were unaccompanied when they went for testing potentially portraying a desire/pressure to demonstrate being in control [45]. The few men who were accompanied to the facility were too sick to reach the facilities by themselves. Generally, disclosure to partners occurred after the HIV test was conducted and test results confirmed.

#### HIV testing experience

Most participants (n = 21) mentioned certain standard procedures prior to and after testing which included being asked for permission to test, as well as receiving counselling before and after testing. The pre–and post–test counselling was well received by a clear majority of men (21/30). Most men (n = 21) were happy with the in-person interactions with providers and as a result, they affirmed embracing a positive attitude towards life and readiness to follow anti-retroviral treatment (ART) schedule.

*First*, *the lady (counsellor) I found here handled me very well*, *she received me very well in a manner that really impressed me*. *Even during testing she encouraged me*. *She gave me a very good example I will never forget*, *she told me there is someone who might have left his house today*, *left things nicely but on reaching the road he got hit by a car and died*, *so I felt a bit relaxed and I accepted*, *I have even gone for classes (counselling) three times*, *the teacher (counsellor) taught us very well*, *I have not seen any issues concerning the services here*. *(PHLIV*, *39 years*, *married*)

However, there were a few men who expressed dissatisfaction with the counselling process mainly due to impatience arising from the “time taken to conduct counselling”.

*I think they (providers) need to be efficient so we can also go back to work*. *For me*, *I call beforehand and ask if I can go at a specific time if they are free*. *I don’t like to sit and wait*. *(MLHIV*, *38 years*, *married*)

### Barriers to HIV testing

In total during our analysis, seven themes were identified as barriers for HIV testing. Analysis showed no major differences in the emerging themes from participants that were categorized as “early”, and “late” testers based on their CD4 cell counts. The identified themes include (1) Perceived providers attitudes; (2) wait time and inconvenient HIV testing clinic times; (3) low perception of risk; (4) limited HIV knowledge: (5) anticipated stigma and discrimination: (6) location and set up of health facilities; and; (7) fear of having a positive HIV test (Table 2).

**Table 2:** Socio-demographic characteristics–counsellors.

Characteristics	N = 8 n (%)
**Formal Education and training**	
O level	8 (100)
College (HIV Counselling training)	8 (100)
**Gender**	
Male	2 (25)
Female	6 (75)
Age—Mean (range) SD	36 (30–50) SD 6.740
Number of years in service–Mean (range) SD	6.1 (2–15) 4.00

#### Perceived negative providers’ attitudes

Prior to visiting health facilities, several participants admitted to being hesitant to visit the health facility to have an HIV test done because of doubts about how providers would handle them due to the information they had heard about the conduct of providers. Mostly, participants had negative information on providers’ behavior and generally this was based on hearsay, own past experiences or their partners’ experiences during childbirth. Thus, real or imagined negative attitudes of providers coupled with the uncertainty of facing an HIV diagnosis made several men hesitate to visit the HIV testing center. The commonly held notion about negative behaviors of health workers was evident from participants’ reference to the health professionals as “rough”, “unkind”, or “time wasters”. The following quotes depict the common view shared by participants.

*Yes*, *I was worried because I always heard that there are some doctors who are always rough and so I was wondering what if I get in there and find such doctor*, *it is not a must I get tested*, *I will look for elsewhere or I may as well drop the idea of being tested*. *(MLHIV*, *39 years*, *married*)

*No*, *that thing is so important because where I went*, *if it were not for those doctors and the way they talk to people*, *you know there are times when you hear that the doctors are not so kind*, *like for example when my wife gets children she will tell you the way the doctors are rude*. *But for those who offer these services are very good*. *(MLHIV*, *30 years*, *married*)

Although participants held negative perception about providers prior to testing, the views changed after the test for many and participants highlighted their satisfaction with services when they accessed HIV testing services and had interacted with the HIV testing staff.

*I used to dislike them*, *and I wondered what they had to tell me*. *I only made my way here because of being ill*, *otherwise I thought they were time wasters*, *little did I know that they were good people with good intentions towards one’s health*, *offering good advices*. *(MLHIV*, *40 years*, *married*)

*But as far as the staff and the center was concerned*, *I had no doubt*, *that anything to do with my status would be leaked*. *But as far as the environment [social relationships] was concerned*, *I would be lying if I said there was no issue*. *(MLHIV*, *24 years*, *Single*)

#### Wait time and inconvenient client hours

The opening and closing times and long queues at health facilities offering HIV testing services hindered men from accessing testing services in a timely manner. The service hours of facilities offering HIV testing services and delays due to high client volume were cited as impediments for testing due to a conflict with work schedules. Typically, most participants work for a 5–6 days (Monday-Saturday) from 8 am to 5 pm with a one-hour lunch break while some have evening shifts when most facilities are closed. Given that men’s work schedules conflict with that of facilities, only a few men can access testing services during the day when most facilities are open. Thus, concerns about work schedules, time availability and access to quality HIV testing services were frequently mentioned as barriers to HIV testing.

*…you can come here and take three hours*. *You should just come pick your drugs and go so if you had work you can go and do it*, *I think that will be better (MLHIV*, *38 years*, *Married*)

*Men are work oriented so with perception they feel like when they come for HIV testing*, *it is something that just involves pricking and result interpretation*. *What makes them unable to get the services appropriately is the time factor*. *They believe it just has to do with pricking and interpreting the results*. *(Provider*, *Male*, *30 years*, *Facility 1*)

#### Low perception of risk

For people to take appropriate actions to protect themselves against HIV infection, they first must perceive themselves as potentially being at risk of becoming infected due to exposure to risk behaviors. However, participant interviews show that some men (9/30) took time to test because they had a low perception of risk and held the belief that they had a small chance of getting infected with HIV–they frequently talked about lacking self-awareness of their risk despite engaging in unprotected sex with casual partners and often under the influence of excessive alcohol consumption, as cited by these MLHIVs.

*…when I was indulging in risky behavior [sex without a condom with a casual partner]*, *most of the time it was in the heat of the moment*. *Okay not most of the time but the only instances when I needed to clear my mind*, *under the influence of alcohol and you know most of the time when such things happen you are not aware or in control*. *(MLHIV*, *24 years*, *Single*)

*Whether I was at high risk or low risk or no risk I would not have concentrated on it*, *seriously*, *because most of the time I used to be intoxicated*. *(MLHIV*, *36 years*, *married*)

Furthermore, health providers maintained that despite repeated exposure to high risk situations, some men didn’t bother to test because they thought they were safe owing to the information they received from their regular sexual partner (s) that those partners were HIV negative.

*…we also get some female or some male who comes and says once… “na si bibi yangu alipimwa” (my wife was tested) why should I get tested*? *… They will tell you … my wife was pregnant [and tested for HIV]*. *I know my wife’s status is my status (Provider*, *Female*, *31 years*, *Facility 2*)

#### Knowledge of HIV

As existing evidence suggests, common reason for hesitating to be tested for HIV centers on limited knowledge of HIV and misconceptions of being infected with HIV [46–48]. While most men (n = 23) knew about basic information about HIV, it was clear that detailed information men had on HIV was insufficient likely due to where they initially obtained the formation (such as from schools, peers, media, and herbalists etc). When probed for what they knew about HIV, most men talked about the life-threatening condition of advanced HIV disease, fear of being infected with HIV and how fatal HIV is without elaborating or discussing critical underlying issues related to HIV testing such as mode of HIV acquisition, transmission, prevention and management, as characterized in the following quote:

*Yes*, *I knew if you contract it you would not even last three months before you are dead*. *I knew it killed very fast*. *I did not have a lot of information*, *but I knew there are medications which people take*. *I also knew it is a killer disease (MLHIV*, *36 years*, *married*)

However, one participant admitted to totally lacking information about HIV and only got to know about it when he visited the health center and got tested. Responding to a question on what he knows about HIV, the participant replied that, *“I was blank*, *I did not know anything*, *I never even thought there is HIV and AIDS*, *I learnt of it after I tested positive”* (MLHIV, 30 years. married).

Most of the information about HIV (both correct and incorrect) was obtained from a variety of sources including school, seminars, and from individuals such as herbalists, medical professionals and musicians. A few men spoke of seeking HIV information from the radio, television or internet after they had developed health problems.

*On the streets*. *Herbalists talk to people on the roads and they also talk a lot about HIV (MLHIV*, *42 years*, *Single*)

*There are those doctors who always passed by (referring to mobile clinics) saying*, *it was better to always know your status*, *how you live*, *if you are HIV positive so that you are able to know how to take care of your family*. *(MLHIV*, *39 years*, *married*)

**Perceived HIV stigma and discrimination** emerged as a key determinant of HIV testing. Despite HIV being in its third decade, there still appears to be high levels of stigma that limit access to HIV services [17, 26, 49–51]. Narratives from participants show they were concerned about what people might say if they found that they were HIV positive. In view of this, some men were concerned about the reaction they would receive from their peers, close relatives, and health providers. They described fears about disclosure of their HIV status, fear of being seen at the facility, including physical appearance due to HIV disease and rumors of deteriorating health. However, self-doubt from negative judgment and shaming emerged as a consistent theme in the narratives.

*I was stressed*. *I imagined what people would say about me and felt like I was dying*. *I thought I would change physically*, *and people would know*. *I thought I would look old*, *skinny and start diarrhea*. *(MLHIV*, *30 years*, *married*)

*It is because people are not good people*. *People will keep pointing fingers and always be warily of you*. *People will not want to mingle with you…Yes*, *even that friend of yours will struggle to be close to you*. *(MLHIV*, *51 years*, *divorced*)

#### Location and set up of facility

Closely related to stigma and discrimination is location and set up of facility which was a key consideration in choosing where to test. Most men reported that they elected to test at a specific facility owing to its “privacy” or for being located “far away” from where people know them. Men kept away from health centers or HIV testing sites which were close to where they lived or where they suspected they might be known to or recognized by health facility staff. Consequently, to identify a suitable health facility, some men ended up incurring transport money to access services deemed private and confidential services.

*For me…this hospital is far from where I live and as I explained to you even if we meet on the streets*, *they won’t expose me …we will just exchange greetings and life goes on*. *I was assured that I can come for medication or treatment any time*. *(MLHIV*, *42 years*, *Single*)

*I heard of that clinic before I knew of my status*. *I heard from a colleague that those ladies who sit outside that clinic usually go to get ARVS [anti-retroviral drugs] and I thought to myself if people have that perception if I go to a clinic near home I will be found out*. *(MLHIV*, *37 years*, *married*)

Coupled with location, the physical structure and set up at the facility was also a key consideration before testing. In general men tended to avoid facilities with isolated testing sites or open examination rooms which they feared could expose them and be recognized by other people at the facility a finding which resonates with previous studies done in sub-Saharan Africa [25, 52, 53].

#### Fear of having a positive HIV diagnosis and access to testing services

For most men the dominant fear was a positive HIV test which appears to be a strong deterrent for testing. Potential negative consequences of a positive diagnosis cited by participants included losing their partner, isolation due to stigma, anxiety and on the extreme end, suicidal ideation. Fears expressed in reaction of receiving a positive diagnosis was regularly expressed by both men and health providers:

*Some men tell you if I find out [I am HIV positive] I will die right here…*. *there was another one last week he said madam if I know my status right now am going to kill myself*. *(Provider*, *37 years*, *Female*, *Facility 3*)

Similarly, the fear of the “unknown” as some providers put it was intricately linked with late entry into care and was often cited as a reason for high mortality among men.

*They have the fear of the unknown*. *I can say*, *some have the fear of the unknown that I get up*, *and just for a testing*? *Ahh*, *that one no…So*, *they fear HIV test*. *They accept when they are told you’re testing malaria*, *you test even the prostrate*, *but not VCT…*. *(Provider*, *Female 31 years*, *Facility 4*)

In addition, health providers frequently talked of difficulties implementing provider-initiated testing and counselling (PITC) strategy (HIV testing routinely recommended by health care providers to persons attending health care facilities as a standard component of provision of services), mostly due to the challenges of convincing men receiving general services to have an HIV diagnosis. Providers found that for the most part some men accessing general health services were uncomfortable and ultimately became resistant when they were advised to undergo HIV testing.

*Okay about that one…*. *you know about PITC you know those ones have come for treatment they have not come for testing*. *Other men even can just come and tell you I am not ready for that please madam*, *so you know you can’t force somebody that they should be tested…so you just tell that person that when you are ready it’s good to come that’s all (Provider*, *30 years*, *Male*, *Facility 1*)

The narratives also suggested that some men believe that if they are in good health, they do not need to undergo the test. One health professional reported that,

“*I don’t know if it is correct*, *but men find it hard to visit hospitals if they are not sick*, *so men are supposed to be persuaded and talked to convince them for testing*. *But it is generally hard for them to decide on their own*”. (Provider, Male, 30 years, Facility 1)

### Facilitators of HIV testing

Understanding factors that facilitate HIV testing can help programs focus on important aspects of HIV testing services. Participants discussed factors that can enhance HIV testing both at the health centers and within the community, however across themes there were minor differences among early and late testers. These factors fall under two categories–individual level factors and health facility factors–and generally focusing on the following themes, some of which have been documented in other studies: (1) High risk perception [6, 14, 36, 54]: (2) Severe illness; (3) Awareness of partners’ HIV status; (4) Privacy and confidentiality [29, 55]: (5) Anticipated quality of services [23]; (6) Availability of providers, supplies and equipment [23]: (7) Flexible/extended opening hours [56]; and (8) Pre–and post–test counselling (Table 3).

**Table 3:** Barriers and facilitators to HIV testing.

Barriers	Facilitators
Perceived providers attitudes^a^	High risk perception^a^^,^^b^
Wait time and inconvenient HIV testing clinic times^a^^,^^b^	Severe illness^a^^,^^b^
Low perception of risk^a^^,^^b^	Awareness of partners status^a^^,^^b^
Limited knowledge^a^	Privacy and confidentiality^a^
Anticipated Stigma and discrimination^a^	Quality of services^a^
Location and set up of facilities^a^	Availability of providers supplies and equipment^a^
Fear of having a test^a^^,^^b^	Flexible operating hours^a^
	Pre- and post-test counselling^a^

**a.** Exclusively expressed by male participants.

**b.** Expressed by both male participants and providers.

#### High perception of risk

Some MLHIV who did not delay or hesitate to test (early testers with CD4 cell counts>500), reported that the motivation for testing was being aware of the risk of HIV infection following exposure to any of the known high risk activities such as unprotected sex, multiple sexual partners, inconsistent condom use. They also felt that having sex under the influence of alcohol placed them at heightened risk of being infected with HIV as illustrated by these men:

*I had many girlfriends and I was not very careful; I was influenced by drinking habits*. *(MLHIV*, *42 years*, *single*)

*“Like for two years before getting tested I was going through some sort of depression phase*. *And throughout this phase I exposed myself to a lot of risky behaviour*, *you know including indulgence in alcohol consumption and risky sexual behaviour*.*” (MLHIV*, *24 years*, *Single*)

Health providers also mentioned that a recognition of their ‘high risk exposure’ (e.g., unprotected sex) was the motivation for seeking a test among early testers. After engaging in a high-risk activity, a combination of worry and fear follows which leads them to get tested, as one provider noted:

*…And*, *some (men)*, *are open …and some say… I know I have been here and there with different partners*. *So am worried*, *am worried*. *I just want to know*, *that’s why they want quick service*. *I want to be tested just to know*. *He is worried and stressed*. *(Provider*, *Male 30 years*, *Facility 1*)

#### Severe illness

Some participants, especially the late testers arrived at a decision to undertake an HIV test after experiencing severe illness. Persistent, and often unexplained illness motivated or forced some men to seek testing to know what they were suffering from. In many cases, with deteriorating health this process was also driven and encouraged by health providers or close family members.

*“I had a problem with my health so they [providers] told me I must be tested for HIV that is when I went and got tested and after that they asked if I would be willing to use the drugs because I was found to be positive*. *(MLHIV*, *31 years*, *married*)

Similarly, providers alluded to the fact that men are reluctant to seek health services. Whereas this was the case, unexplained and prolonged illness, as well as suspicion of having a sexually transmitted infections (STIs) drove men to seek health services including HIV testing.

*“…you know most men when they feel very ill is when they go to the hospital but if they are not very ill you won’t see them in the hospital*.*” (Provider*, *female*, *35 years*, *Facility 5*)

*Most of them… or some of them it is the illness maybe like they have a headache or stomachache which makes them to come by the way*. *And*, *here in [Health facility name] we also have the STIs they are making people to come so much*. *If one has an STI they feel like if I have an STI I must be having HIV*, *so you get them coming more often by the way*. *(Provider*, *female*, *41 years*, *Facility 6*)

#### Awareness of partners HIV status

Participants felt that knowledge of a partner’s HIV status was a catalyst for testing. Regardless of how participants became aware of their partner’s HIV status–whether through direct disclosure by the partner or through their own suspicions about the partner’s health/status–almost always this was followed by a sense of urgency to go and test.

*It’s because I learnt that my partner was positive and that we are actively engaged in sexual activity*, *and so I thought there was no reason for me to shy away from this and thus I convinced myself that I needed the testing”*. *(MLHIV*, *51 years*, *married*)

In addition, providers emphasized that men were more inclined to test when they entered a new, temporary or permanent relationship.

*“… Some who are married come because they want to…because he’s being troubled by the wife*. *And so*, *I want to get another wife and since I have children or my first wife died*, *I don’t want to bring them a sick mother*. *So*, *they come for testing because most them*, *for them to come*, *I told you they have a reason*. *You see*, *so many of them*, *they fear HIV*. *(Provider*, *Female*, *31 years*, *Facility 7*)

#### Confidentiality and privacy

Two aspects of privacy and confidentiality were noted. First, participants suggested that health facility staff should be professional and always endeavor to conceal patient information. This issue emerged out of the recurrent concerns raised about unexplained and negligent disclosure of client’s HIV status to other people. One participant said:

*“I was at [Name of facility] where the doctor accidentally broke the news to my mom*. *It would really help if the hospitals first asked rather than saying it out aloud*. *I am so fortunate to have understanding parents and people around me*. *Perhaps if things were a bit different saying it out aloud would not have been an issue for the patient*. *Confidentiality is an issue*.*” (MLHIV*, *24 years*, *Single*)

Second, participants felt that testing sites should be in discreet locations that can best protect their privacy. Several men as well as providers suggested the need for an enabling environment in facilities that inspire client’s confidence to access HIV testing services. Participants raised concerns about easily identifiable locations for offering HIV services within facilities that lacked both visual and audio privacy.

*Okay in my own opinion I think it is all about the privacy that can make one prefer other options*, *because when you get to that room everyone will know this person is infected” (MLHIV*, *25 years*, *Married*)

*For example*, *for this place the room that we go to is next out-patient area so you will find that we are even sharing the same bench with them so when you get to the room other patients just know you are going for HIV services and people will just starting talking about you going to HIV center*. *(MLHIV*, *25 years*, *married*)

#### Anticipated quality of services

Clients views were sought on testing services and what they thought were the best ways for health facilities to promote HIV testing and treatment services for men. Based on responses to this question, three key aspects emerged highlighting important features of service delivery. These were quality, privacy and friendliness of services, which participants felt were critical in the choice of a facility.

*Friendly services mean personalized services and the service providers see you as a friend*. *It is a place where you are confident that you will get the services that you want*. *I even told my wife to stop going where she goes because they are made to line (queue) up in the open where everyone can see them*. *I even brought her here because they are keen in maintaining confidentiality and not put on a bench like rubbish*. (MLHIV, 37 years, married)

Further, participants’ interpretation of quality of services entailed the whole spectrum of services provided at facilities from initial contact with staff at the reception area, interactions with providers in different departments, to availability of HIV-related expertise.

*In my opinion*, *friendly services would mean*, *that the moment a client comes in*, *one way you can help such a person would be even in a case when they have come when they are very sick*, *you know at times there are people who can come in when they are really in a bad state health-wise*, *then you hear the doctor asking*, *“where were you that you waited too long to look like this*, *how are we supposed to help you*?*” You realize there someone may lose hope*, *because they will be telling themselves*, *you see even the doctors have seen it that I will not make it even if I am given the drugs*, *so there is really no point of taking the medication* …*” (MLHIV*, *39 years*, *married*)

*“I would say when you walk to any health institution the first impression is at the reception before you even go to see the doctor and explain your problems*, *like if I have to get medicine and the receptionist is rude the distance starts with the first stage which is the receptionist then goes up to the doctor*. *What is the reaction of the doctor*? *How does he take it*? *How does he advice you*, *yeah*?*” (MLHIV*, *24 years*, *Single*)

**Availability of supplies, providers and equipment** was also reported as an important factor influencing access to and motivation for HIV testing among men.

*“It (health facility) should have plenty of kits*, *drugs and enough doctors so that patients do not queue for long*. *It is not good when they are few*. *Doctors are there but there are no medicines and even for other illnesses” (MLHIV*, *37 years*, *Married*)

#### Flexible/Extended opening hours

As noted earlier, current service hours at health facilities prevent working men from accessing testing services due to their work schedule. Almost all participants suggested that operating hours at the facilities should be made flexible to accommodate their tight work schedules with proposals for facilities to provide an all-round 24-hour service to address the twin concern of conflicting work schedules and the provision of discreet services as cited below.

*…they should make these hospitals operate 24 hours because this one is a big hospital*. *The reason why I’m saying that is because there are some people who work in daytime up to very late and when they come here they don’t get services because the doctors and the nurses are gone*. *(MLHIV*, *40 years*, *married*)

*“I guess night shift*. *At least some guys could be coming and popping in and you know most of the guys do not want to be seen entering this hospital and maybe some are working*, *for instance*, *Saturdays and Sundays*. *(MLHIV*, *25 years*, *Single*).

#### Pre- and post- test counselling

Standard pre- and post- test counseling services provides information on the technical aspects of testing and the possible personal, health, social, and psychological implications of being diagnosed as either HIV positive or HIV negative. Participants felt that counselling should extend beyond this and include instilling confidence to disclose their status to their partner, enhanced couple testing, enabling clients to cope with the HIV diagnosis and preparing individuals for ART initiation.

*It is helpful*. *If it was not for the counselling*, *I had decided to chase away my wife*. *They told me to take it easy and I can live positively*. *I was only thinking about death*. (MLHIV, 37 years, Single)

*Before I was tested*, *I was counselled by the doctor and by that time I decided that if I turn out positive*, *I will just take it normal*, *I will start medication and I will follow the doctor’s advice*. *You must continue with life*. *I believe it is not the end*. *He really helped me because by the time he asked me whether the result would be positive or negative*, *I just accepted*. *(MLHIV*, *51 years*, *divorced*)

## Discussion

To date, in Kenya a large body of research on HIV testing among men has focused on the quantitative measurement of PMTCT [57–60], male involvement [61–63], and oral self-testing [64–66] with limited data on standard HIV testing offered within regular health care settings. Studies utilizing qualitative approaches to document insights and perspectives of men on HIV testing experiences are lacking in Kenya. Yet, such data offer rich lived experiences that can better provide the context and experiences of HIV testing and potentially help improve services. This paper utilized in-depth interviews with MLHIV and HIV testing counsellors to understand the drivers of HIV testing among men in the city of Nairobi. There are several strengths of this study. First, we conducted the interviews with participants who were recently diagnosed with HIV and were on ART (participants were classified as “early” and “late” testers based on their CD4 values) many of whom during interviews recalled their recent HIV testing experiences (6–12 months ago), rather than querying hypothetical experiences to document their positive deviance behaviors. Second, we conducted the study in health facilities with differing characteristics (facilities with high and low client load, high and low proportion of male versus female testers and different catchment area i.e. low income vs. high income area, industrial area vs residential, etc., and HTS type–i.e. sites where antiretrovirals are offered versus free-standing HTS sites) and in a setting with a moderate to high burden of HIV among the general population, which allowed us to elicit rich experiences around HIV testing that possibly influence HIV testing decision–making process. Clients accessing HIV testing services are confronted with different challenges (stigma, providers attitudes and distance, etc) in deciding where to take an HIV test and therefore by documenting clients perspectives from diverse settings we provide a nuanced understanding on men’s perceptions and reasons for testing at the facilities. Third, the study obtained data from both MLHIV and HTS counsellors ensuring triangulation of themes. Hence, to design targeted interventions aimed at improving uptake of HIV services, it is essential to have a nuanced understanding of men’s experiences with HIV testing services–the context, barriers and facilitators–from clients and providers.

Our findings highlighted several barriers and facilitators to HIV testing among men. Most of the key barriers to testing were at the individual and health facility level which largely intersected with stigma and discrimination and likely affected timely access to HIV testing services. In addition, with the HIV epidemic in its third decade in the country, the assumption was that knowledge of HIV testing was near universal and widely accessible everywhere, yet, our findings showed that critical knowledge gaps still exist thereby delaying HIV testing and entry to care. Our findings are consistent with other studies in Africa, [67–69] that further show that the risk perception of HIV infection was low among some men as they did not view themselves to be at risk even when engaging in high risk behaviors or altogether lacked self-awareness of risk due excessive consumption of alcohol. In their study, Velloza et al. [70] found that individuals who used alcohol had twice the odds of ART non-adherence compared with those who did not use alcohol (34% non-adherence among alcohol users vs. 18% among non-users; pooled odds ratio: 2.25; 95% confidence interval: 1.87–2.69; p < 0.001). While prevalence of alcohol misuse based on AUDIT/CAGE in SSA is estimated at 32.8% (IQR 20.8–48.5%) [71] and 31.7% (95% confidence interval (CI): 26.8%–37.2%). In Kenya, it was found that the prevalence rates for alcohol consumption was higher in men (54.6%) than in women (8.9%) [72]. The inability to identify exposure to risk or downplaying exposure to risk can have far reaching consequences to an individual or their sexual partner (s)’ health due to repeated exposure to risk arising from lack of knowledge or agency to seek services. As a result, HIV programs need to actively incorporate alcohol related interventions (e.g. screening, messaging and counselling) to mitigate the effects of alcohol on personal risk especially helping men better assess their risk of HIV.

A common view among participants was that most working men find it extremely difficult to access HIV testing services during normal working hours due to conflict with work schedules. This issue is amplified with the desire to seek services far away from areas of residence. Thus, a sizeable number of working men may miss out on critical HIV testing services in health facilities owing to work commitments unless they identify facilities with flexible operating hours are convenient for them. Men’s work commitments may also affect their ability to use community-based HIV testing services. Our findings are consistent with research findings from other sub-Saharan African countries, including Zambia, South Africa and Burkina Faso where overall HIV testing rates for men is disproportionately lower than among women [10, 11, 73]. This suggests that men in the SSA region may face similar barriers to HIV testing at different levels including at the individual, health-care system, and interpersonal levels where perceived negative provider attitudes, location and set up of health facilities, inconvenient operating hours, and low perception of risk create barriers to HIV testing and increase the risk of HIV infection [9, 12, 17, 25, 50].

More so, our findings show that site location, setup of facilities, perceived providers attitudes, privacy and confidentiality overlap with a strong sense of anticipated stigma. Often, participants were conscious of provider attitudes especially where and how facilities were set up mainly due to concerns around their privacy and confidentiality which points to deep rooted fear of testing or being identified to be HIV positive. Stigma and discrimination has been found to affect access to health services and non-disclosure of health conditions owing to fear of being isolated or rejected which may result in non-adherence to or low uptake of medical advice [49]. Our findings document several instances where participants’ access to testing services was delayed by concerns around stigma and discrimination. Although numerous national campaigns to promote HIV testing in Kenya have been implemented countrywide in the past it appears stigma is still pervasive and continue to hinder access to testing services. Stigma and discrimination should also be addressed nationally and within the communities and at the health centers as it appears stigma in all its forms is given less emphasis in HIV programs. This likely points to a gap in effective strategies to address stigma and discrimination, without which the attainment of the 90-90-90 country targets might be in jeopardy. A review of a study conducted in South Africa more than a decade ago showed that HIV-related stigma drives the pandemic out of the public view, a phenomena which is still occurring among clients seeking HIV-related services [26]. Similarly, evidence from other settings [8, 18, 74, 75] demonstrate that stigma, discrimination and fear of having a positive HIV test result are critical factors for both testing as well as linkage and retention in HIV care among men [17, 50].

Interestingly, despite uncertainties of how providers would handle them, there was a significant change among MLHIV perceptions about provider attitudes before and after undergoing HIV testing and related counselling. Prior to HIV testing, it appears negative perceptions about provider attitudes impeded the uptake of testing. However, these perceptions appear to improve due to the quality of interactions with providers and services received. Thus, programs should use the positive client experiences to enhance post-testing actions including enhancing referrals and linkage to care and other support services. However, in their findings from providers interviews in Western Kenya, Genberg et al. [76] found that health system factors impact patient engagement in HIV care due to work environment that constrained providers ability to deliver high-quality HIV care and encouraged negative patient-provider relationships. Studies among other key population groups such as men who have sex with men have shown that access to HIV care is impeded by HIV-related stigma, lack of access to friendly health services, economic and social challenges due to stigma, difficult relationships with care providers, and discrimination at the clinic and in the community [77].

The in-depth interviews with men and counsellors identified several facilitators for the uptake of HIV testing: high perception of risk, severe illness, partner’s HIV status at the individual level and perceived quality of services including role of counselling, facility opening hours and availability of privacy and confidentiality; and availability of providers, supplies and equipment at the facility level. Content identified in the themes in the Kenyan context, and specifically relating to male testing that have not been well documented in previous studies include *severe illness*, *knowledge of partners status* and *the role of pre-and post-test counselling* in facilitating testing and enhancing coping mechanisms and linkage into care. Further exploration is needed to assess reasons for men’s delay in seeking HIV testing services and the context of prolonged ill health. Strategies to enhance disclosure of HIV status to partners is needed both at the individual and health facility level to improve couple counselling and HIV testing in general. Although pre-and post-test counselling largely provides information on the technical aspects of testing, our findings demonstrate that its benefits go beyond the mere provision of HIV-related information to shifting attitudes and enhancing early initiation to treatment and subsequent linkage and retention in care.

Most experiences of early and late testers overlapped across themes implying that the challenges men face before, during and after testing might be cross-cutting among different categories of men. However, further exploration of the barriers to testing is needed to objectively identify their correlation with HIV testing. Moreover, in view of our findings, there is need to address gaps in risk perception, HIV knowledge and access to accurate information, couple communication, stigma reduction and health awareness and agency to access health services. A focus on understanding the facilitators for HIV testing is also needed to better understand how these factors can be amplified/applied in HIV programs since the design of HIV testing interventions can be context specific.

There are a few study limitations to note. Given that many participants were recruited from the health facilities it is possible that these participants face fewer barriers (or possess unique attributes that enhance testing–positive deviance) to accessing HIV prevention and sexual health services compared to those in the community who do not test or those with no or limited access to services. Also, our sample size is small (30 men) and the perspectives of men presented in this article might not be representative of all the men accessing HIV care at health facilities in Nairobi and Kenya in general. Further, given the limitations of qualitative inquiries and the fact that sampling of participants focused on specific sub-counties in Nairobi, our findings may not be generalizable to all men in Kenya and hence should be interpreted with caution.

In conclusion, owing the importance of HIV testing in HIV prevention and the fact that it provides an important entry point for care for persons newly diagnosed with HIV, enhancing access for HIV testing services, especially for men, is a critical first step in linking them into care and addressing the HIV epidemic. Our findings identified multi-level barriers and facilitators for uptake of HIV-testing and provided a summary of implications in public health practice. HIV programs need to address the context, barriers and facilitators for testing to increase uptake of testing and to link MLHIV to care. The findings also suggest that it may be prudent to revisit policies and guidelines relating to testing and treatment with a view to aligning them to with some of the gaps and opportunities identified in our findings and other related research. For example, PITC may be effective when men present themselves for treatment during severe illness, but there is a need to specifically address men’s apparent poor health seeking behavior and specific behavior relating to uptake of HIV testing and treatment services. Also, there is a need for more robust studies to investigate the complex interactions of the barriers and facilitators for testing in the current context in the country, if Kenya must meet its 90-90-90 strategic goals.
